# Dr. Wood, in Reply to Dr. Kentish

**Published:** 1808-06

**Authors:** James Wood

**Affiliations:** Newcastle upon Tyne


### *To the Editors of the Medical and Phyfical Journal.*

Gentlemen,

Dr. Kentish, in his notice of my claim, vid. Med. Journ. No. 109, p. 30, has laid himself very open to animadversion and retort from me; but I will avoid both, as much as the necessary statement on the subject will allow me.

My

My assertion may be divided into two parts. 1st. That I could produce strong circumstantial evidence that the following words, (in a publication* of mine in 1793,) "we can apply not only with safety, but with the best effects, ardent spirits to a part burned or scalded," were the origin of the stimulating plan of cure by Dr. (then Mr.) Kentish in this place, and of his consequent publication on the subject. 2d. That this hint, for such treatment of burns and scalds, was the first made here, and that I had always openly maintained its *priority*.

It will not be requisite to bring into one point the whole of the circumstantial evidence I have alluded to, it will partly be collected in the remarks I have to make, and partly, in the proofs I bring forward of my *priority, which,* indeed, *alone,* it seems necessary to substantiate to the tribunal before which I have made my claim.

On the 4th of September, 1792, the publication, containing the words and principles on which I make my claim, was read *in manuscript* before the Medical Society of this town, which manuscript was afterwars circulated amongst the members of it, (then almost all the medical gentlemen of the town,) at their own houses, and by their request. Not quite three months after, viz. in the November following, Dr. (then Mr.) Kentish, dates (the stubborn fact he mentions,) *his "first recited case,* in which the whole of the third, or *complete method of treatment* is delivered."

The manuscript I have mentioned was printed, and it was published in Feb. 1793. Dr. (then Mr.) Kentish's *first Essay,* appeared in 1797, so that here is clear proof and demonstration of my *priority* in *private* and in *public.*

Now, Gentlemen, Dr. Kentish says, "*upon what principle* Dr. Wood is to substantiate before a Court of Justice that my practice in 1792 was *influenced* by a publication of his in 1793, I am perfectly at a loss to divine; but as he has made the assertion, the task must be left to him." The task, therefore, is very easy to me, for I have only to answer, *upon the simple principle of the above dates,* viz. that my *manuscript was read and circulated in Sept.* 1792, *his practice began* in Nov. 1792; my publication was in 1793, his in 1797.

The great point, *priority,* being thus substantiated, I

* Entitled, "Thoughts on the Effects of the Application and Abstraction of Stimuli on the Human Body, &c."

must now reply to some particulars, mentioned by Dr. Kentish, of a collateral nature. Dr. K. says, "does not Heister give me as much information on the subject as Dr. Wood?" "Sydenham, above a century before, was certainly as minute in the manner, as he was decided in the advantage to be derived from the application of spirit in burns, as Dr. Wood was in 1793; but had the ideas of Heister, or of Sydenham, produced *any plan or system* which philosophy could regard as dictated by common sense, *until the period when I wrote my Essay*?" Yes, for I did, at the same time, produce *a plan or system*, which Dr. Kentish's philosophy at least approved, because, *in his first recited case* he delivers the whole, or the complete method of treatment, on the very principles of the system supported in my manuscript and publication; which also afforded *something* "to guide the choice of the inquirer" amongst "the labyrinth of opinions on this subject," *some* "principle to *lead* the practitioner," *before* the time Dr. K. wrote his Essay. In my publication, therefore, Dr. Kentish might have been fortunate enough to *find a "principle* to guide the practitioner to give relief;" and a system, in which "every thing was" not "full of doubt." Dr. Kentish's question, then, "I should be glad to know upon what principle Dr. Wood supposes his idea should produce so much, when the ideas of at least as great men as himself should have produced no effect at all?" is sufficiently answered.

You will perceive, Gentlemen, exactly the nature of the claim I made; but Dr. Kentish has put it into stronger terms; he has laid the indictment against himself, in which he is charged with "Literary Piracy," and an "unprincipled suppression of the source from whence he drew his information;" and against this high voluntary indictment, he pleads not guilty, by saying, "I here solemnly assert, that I never saw, read, or heard of *the passage* * alluded to by Dr. Wood *until after* I had published my first Essay;" that is, Dr. Kentish never saw, read, or heard of the passage in my publication in 1793, until after his in 1797. To this I will only answer, that if Dr. Kentish could produce a *certificate* that he could not see, read, nor hear, from

1793

* It will be observed, that Dr. Kentish only solemnly denies having seen this particular *passage*, not the principles or system of which *it* was given as an example. Yet Dr. Kentish says, before that a little, that there was no plan nor system in existence in 1793, nor for four years after, until 1797, *the period when he wrote his Essay.*

1793 to 1797, still even that would not, *could not*, set aside my claim of *priority* in the *judgment of the public*, as my publication *was to be* seen, read, and heard of, during that period.

It is, I think, necessary to take some notice of my *sleeping* justice. I will not remind Dr. Kentish of my *unreserved* and constant conversation on the general principles which I published in 1793; I will not remind him of the time and place when I met him the first time after his publication in 1797, and when he asked me, whether I had read it, and that I said I had, but that there was nothing new in it to me, for he would find the practice and principles he recommended in my publication in 1793, p. 45; I will not remind Dr. K. of this, I say, but allow my answer to *sleep* a little longer. I will only inform him that my *sleeping* justice *awoke*, even *in print*, six years ago, in a pamphlet entitled, "A Third Address, &c." pretty generally circulated in this town, and in many parts of the kingdom; this was allowed to *sleep* also; if, therefore, Dr. Kentish *complains* of my *sleeping* justice, I may ask, why did he not try to rouse it from its slumbers? But it appears to me that some other of Dr. Kentish's powers have been liable to slumber: why did his Fluor Volatile Alkali only awake once or twice from 1788 to 1790, and from that time *sleep* until its resurrection in Nov. 1792, in *a new form and spirit*? This transformation exceeds any thing in Ovid's Metamorphoses! Perhaps, Dr. K. may compare the change to that of the chicken from the egg; but then, how did it happen that it was not rightly hatched during the long incubation from 1788 to 1792? Did my *cold hint* in the September warm it, and produce more in three months, than three years could effect without it? Perhaps, if there had not been some warmth reflected from the doctrine I had then thrown out, Dr. K. might have *sat* on the Fluor Volatile Alkali till this time, without incubation producing any *quickening* effect.

But, to be again *serious*. Dr. Kentish does not know whether he may not now rank me amongst those who recommend cold water. In answer to this, I have only to say, that my sentiments are before your readers, and will not be misunderstood. I will, however, take also this opportunity of saying, that, as far as I at present know, I am the first who has pointed out, that there is *one* * state of inflammation

* The state of inflammation opposed to this, is that arising from direct debility, and which only admits of one method of cure; the state of gangrene, inflammation, and of the system, capable of being cured by *opposite* methods of treatment; and that, if this explanation leads to such a scientific view in medicine, as to solve, on fixed principles, questions before inexplicable, to reconcile apparent contradiction, and to lessen disputes among medical men, I shall have afforded a light, to dispel the mists on the subject, that immediately involved us, and to conduct us correctly and clearly a little farther on our way.

I am, &c.

JAMES WOOD.

*Newcastle upon Tyne,*
*May* 10, 1808.

gangrene, into which both kinds of inflammation is liable to go, will require some modified plan of treatment, according to the nature of the previous inflammation.

